# Study on nanocellulose isolated from waste chilli stems processing as dietary fiber in biscuits

**DOI:** 10.1371/journal.pone.0281142

**Published:** 2023-01-27

**Authors:** Yongjie Ma, Xuyan Chai, Hongliang Bao, Yishuo Huang, Wenbin Dong

**Affiliations:** 1 College of Food and Drug, Luoyang Normal University, Luoyang, Henan Province, China; 2 Mathematical and Sciences College, Luoyang Normal University, Luoyang, Henan Province, China; 3 College of Food and Biological Engineering, Shaanxi University of Science and Technology, Xi’an, Shanxi Province, China; Qatar University, QATAR

## Abstract

In order to expand the high added value of waste chilli stems and the recycling of green resources, cellulose in chilli stems was extracted by nitric acid–ethanol method, and nanocellulose was prepared by sulfuric acid hydrolysis method. The results showed that the cellulose content was between 15% and 34.5%. Under the optimum experimental conditions of 60% sulfuric acid concentration, 60°C reaction temperature and 120 min reaction time, the average yield of nanocellulose was 36.42% ±1.36%. Prepared cellulose and nanocellulose had been characterized using scanning electron microscopy, fourier-transform infrared, and x-ray diffraction analysis. The research indicated that the biscuits with acceptable overall quality could be prepared by using the dosage of nanocellulose (7%), and the corresponding biscuits had regular appearance and relatively smooth surface. The total dietary fiber content was positively correlated with different nanocellulose content. Through mice experiments, it was found that the consumption of biscuits containing nanocellulose could significantly reduce the food intake of mice and inhibit the weight growth of mice. Therefore, the research showed that whole wheat biscuits with nanocellulose could be regarded as food rich in dietary fiber. These results provided a basis for exploring the green resource recycling of chilli stems in food processing.

## Introduction

The abundant amount of straw generated during processing chilli has become a large source of vegetable waste. Currently, most waste chilli stems (CS) is discarded, and a fraction of them is burned and returned to fields. However, the allelochemicals generated during the decomposition of CS can inhibit the growth and development of crops. Therefore, it is not suitable to return them to fields [1]. The conventional discarding method involves burning CS and returning them to fields. Although this approach can eliminate the effect of allelochemicals on crops, it causes air pollution. Most CS in China has not been used and is stacked disorderly in fields. CS does rot naturally or have been discarded as waste. Eco-friendly treatment of CS and plant straw has attracted considerable attention. Moreover, many scholars have focused on developing innovative methods for using CS for various applications [2–5].

Straw contains abundant cellulose, which is a natural polymer and a biomass resource with excellent properties, such as high degradability and outstanding renewability [6, 7]. The conversion of cellulose extracted from raw materials into high value-added chemicals is a popular research topic worldwide. Moreover, the conversion of cellulose into nanocellulose presents remarkable application prospects [7, 8]. The primary components of the cell wall of CS are cellulose, hemicellulose, and lignin. However, because these components are intertwined through different binding forces, it is challenging to separate cellulose from hemicellulose and lignin. Cellulose molecules are connected via hydrogen bonds and form bundles. Therefore, because 70% of cellulose is present in the crystalline form, it is difficult to depolymerize it [9, 10]. Nanocellulose exhibits numerous excellent properties, such as natural regeneration ability, biodegradability, biocompatibility, high strength, high specific surface area, high aspect ratio, and good thermal stability, and great application prospects in the biomedical, pharmaceutical, material science, cosmetics, and papermaking industry [11]. Recently, nanocellulose has been used in the food industry as a kind of food thickener, fat substitute, and stabilizer. Studies have demonstrated that nanocellulose can inhibit the growth of ice crystals and improve the taste of ice cream [1]. Therefore, many researchers have focused on expanding the applications of nanocellulose.

There are many methods to prepare nanocellulose, mainly including chemical methods (such as liquid acid hydrolysis, solid acid hydrolysis and TEMPO oxidation), physical methods (high-pressure homogenization, grinding, freeze grinding and electrostatic silk prevention), biological methods (enzymatic hydrolysis and biosynthesis) and so on [12–18]. Although the above methods can be used to prepare nanocellulose, they all have certain advantages and disadvantages, as shown in Table 1.

**Table 1:** Comparison of advantages and disadvantages for different preparation methods of nanocellulose.

Method	Advantage	Disadvantage			References
Solid acid hydrolysis	Mild reaction conditions and less waste discharge.	The reaction efficiency was low, the reaction time was long, and complex pretreatment was required.			[12]
Hydrolysis with hydrochloric acid or phosphoric acid	High thermal stability.		High production cost and poor dispersion in water.		[13]
Organic acids (oxalic acid, maleic acid or toluidic acid, etc.)	The organic acid solution was recyclable and had little corrosion to the equipment.		Weak acidity, low reaction efficiency and product yield, requiring complex pretreatment.		[14]
Catalytic oxidation method (TEMPO, ammonium persulfate)	Good dispersion.		Low thermal stability, relatively expensive reagent and difficult to reuse.	[15]	
Enzymatic hydrolysis	Green and environment-friendly, low consumption of energy and chemicals		Low yield and high cost.	[16]	
Bacteriological method	High crystallinity.		Complex process and high cost.	[17]	
Ionic liquid	Good performance for fiber swelling or solvent.		The purification process was complex, the cost was high, and the thermal stability of the product was low.	[12]	
Mechanical method	Less reagent residue.		Large energy consumption and high cost.	[18, 19]	
Low eutectic solvent (choline chloride, oxalic acid, formic acid or p-toluenesulfonic acid)	Low solvent cost, low vapor pressure, low toxicity and biodegradability.		The swelling and dissolution properties of cellulose were ordinary, requiring complex secondary treatment.	[20]	
AVAP method (ethanol and sulfur dioxide)	High crystallinity, recyclable chemicals, low production cost.		Ethanol and sulfur dioxide pulping were dangerous and toxic, and the equipment was required to be airtight.	[21]	
Sulfuric acid hydrolysis	Simple operation, low cost, mild preparation conditions and wide application.		The recovery and treatment of residues needed further research.	[22]	

In this study, sulfuric acid hydrolysis method was used to prepare nanocellulose. The nanocellulose obtained by sulfuric acid hydrolysis method was suspended in the aqueous solution and was not easy to gather. It was widely used in industry due to its simple operation. However, the sustainable application of by-product recovery in environmental, economic or product performance needs further research. At present, the raw materials for preparing nanocellulose were mainly cotton, wheat straw, rice straw and corn straw. Due to the influence of cultivation area and planting conditions, the raw materials of nanocellulose were limited. However, the cellulose content in CS was up to 37%, but it had not been fully utilized. Therefore, the preparation of nanocellulose from CS in this study had more practical value.

With the deepening of people’s understanding of healthy consumption and whole wheat food, whole wheat biscuits are gaining recognition and attention. At present, whole wheat biscuits in the market often add a large amount of oil and sugar, which has a great negative impact on blood glucose [23]. This processing method has offset the advantages of whole wheat raw materials. In addition, the content of wheat flour in many whole wheat biscuits is much higher than that of whole wheat flour, which can not meet the high fiber expected by people.

To improve the green resource recycling utilization of CS in food processing and meet consumer demand for healthy foods, researchers have focused on in-troducing dietary fiber in baked food products [24, 25]. In this study, CS was used as a raw material. The cellulose in CS was extracted using nitric acid–ethanol method, and then nanocellulose was prepared from cellulose using sulfuric acid hydrolysis method. The prepared nanocellulose was used as a new source of dietary fiber during biscuits processing, and its effects on the physicochemical and functional properties of biscuits were investigated. The structures of CS material, cellulose and nanocellulose were analyzed by scanning electron microscope (SEM). The functional groups of the samples were analyzed using Fourier-transform infrared (FTIR) spectrometer. The crystal structures of cellulose and nanocellulose extracted from CS were characterized by X-ray diffraction (XRD) analysis. In recent years, with the progress of production technique and the gradual promotion of application fields, nanocellulose has been deeply developed in new application fields (food carriers, food packaging additives and food processing), and its scale level has gradually reached the level of large scale production. As the production cost of preparing nanocellulose by sulfuric acid hydrolysis is low, the market demand increases significantly, and the yield of nanocelulose also continues to increase, which is expected to reach 9000 T/a in 2024 [26]. The preparation of nanocellulose from CS is not only cheap, but also conductive to the deep processing of agricultural products and waste disposal. We believe that our results can provide a reference frame for the efficient recycling of CS.

## Materials and methods

### Reagents and materials

Whole wheat flour, coarse grain flour (rye flour, buckwheat flour, sorghum flour), skimmed milk powder, resistant dextrin, skimmed milk, maltitol, baking powder, baking soda, plant oil and salt used in the experiment were purchased from Xinxiang Xinliang grain and oil processing Co., China. Fehling reagent, sodium hydroxide, ethanol, potassium sodium tartrate, copper sulfate, hydrochloric acid, isopropanol, diethyl ether, nitric acid, 95% ethanol, sulfuric acid, and phenolphthalein were bought from Tianjin Fuchen chemical reagent factory. Chilli stem collected from chilli industry, Jilin, China. Nanocellulose was made in the laboratory. All reagents used in this study were analytically pure. Double-distilled water was used throughout the study. Whole wheat biscuits were referred to as “biscuits”.

### Extraction of cellulose from waste chilli stems

Concentrated nitric acid (40 mL) was added to a beaker containing 160 mL of absolute ethanol. The mixture in the beaker was allowed to rest, cooled to room temperature, and then transferred to a brown reagent bottle. The particle size of CS after crushing pretreatment and the times of nitric acid–ethanol extraction would affect the quality of cellulose extraction. In the process of intensive physicochemical treatment, part of the fiber in CS was hydrolyzed into glucose, which would generate dark insoluble matter under the action of hot acid. Through a large number of experiments, it was found that the particle size of CS was 50 meshes, and the extracts turned white after being treated with nitric acid–ethanol method for 4 times. CS was added to a reagent bottle and boiled continuously. The hydrogen ions in the mixture destroyed the hydrogen bonds between cellulose, hemicellulose, and lignin at high temperatures. Lignin was directly oxidized to nitrated lignin using nitric acid, and the nitrated lignin was dissolved in an anhydrous ethanol solution. Hemicellulose was also oxidized and hydrolyzed using nitric acid. Ethanol medium could reduce the hydrolysis and oxidation of cellulose by nitric acid. Therefore, the quality and purity of extracted cellulose was high.

### Preparation of nanocellulose

The obtained cellulose was dried in an oven at 100°C, sieved using an 80 mesh sieve, and stored in a dryer before further use. Cellulose (0.2 g) was added to a 250 mL round-bottom flask, and it was placed in a constant-temperature water bath. 20 mL of sulfuric acid solution was slowly added to the flask, and the mixture in the flask was stirred continuously using a magnetic stirrer. After 120 min, the flask was removed from the water bath, and the reaction mixture was diluted by adding 10 times the volume of double-distilled water to end the reaction. The mixture was centrifuged with 8000 r/min for 10 min, and poured out the supernatant to remove the acid in the supernatant. Double-distilled water was added to the flask again, and the mixture was centrifuged repeatedly until the supernatant obtained after centrifugation was a turbid suspension. The turbid suspension was added to a dialysis bag, and the dialysis was performed using double-distilled water in a 1 L beaker over several days until the pH of the dialysate was neutral. The mixture in the dialysis bag was filtered through a microporous filter membrane, and the substance retained on the microporous filter membrane was dried for 48 h to obtain nanocellulose powder.

### Characterization of cellulose and nanocellulose

The morphology of CS material, cellulose and nanocellulose was analyzed by SEM (Hitachi SU-70 analytical type). The functional groups of the samples were analyzed using a NEXUS-670 FTIR spectrometer in the wavenumber range of 4000–500 cm^-1^ at a resolution ratio of 2 cm^-1^ and the potassium iodide slice method. Potassium iodide was used as the reference. The crystal structures of cellulose and nanocellulose extracted from CS were characterized by XRD analysis (Japanese science Ultima IV) using an instrument equipped with Cu Kɑ radiation (λ = 0.154 nm). Scanning was carried out in the 2θ range of 10–80° at a scan speed of 5°/min. Crystallinity index was calculated according to a method described in the literature [13, 15]. The equations were as follows:

$$CI=\frac{I_{002}-I_{am}}{I_{002}}\times 100\%$$ (1)

where *I*_002_ and *I_am_* are the maximum diffraction intensity of 002 plane and diffraction intensity of amorphous region, respectively.

### Biscuit production

The biscuits recipe consisted of nanocellulose (5%, 7% and 9%), whole wheat flour (50 g), coarse grain flour (30 g), skimmed milk (30 g), resistant dextrin (10 g), skimmed milk powder (10 g), maltitol (15 g) and plant oil. The cooled skimmed milk was added to the weighed nanocellulose powder for emulsification, and the sifted whole wheat flour, coarse grain flour (rye flour, buckwheat flour, sorghum flour), resistant dextrin and skimmed milk powder were added respectively. Stir with a baking shovel to make broken dough, and then knead it into uniform and smooth dough. Wrap the prepared dough with fresh-keeping film, let it stand for 15 min, and let the baking powder and baking soda play their role to make the dough slightly expanded and soft. Knead the static dough again, and discharge some tiny bubbles to make it mix more evenly. Lay silicone paper on the top and bottom of the dough and roll it into about 2 mm thin slices, which should be flat, smooth and consistent in thickness. Press out the figure with a mold and place it in a baking pan covered with silicone paper. They were heated for 16 min in a baking oven, with upper and lower temperatures of 170°C and 165°C, respectively. Then, the biscuits were cooled to room temperature and packaged. For experimental analysis and sensory evaluation, the biscuits were crushed to fine powders, stored in a refrigerator at -20°C and made on the same day.

### Sensory evaluation of biscuits

Through quantitative description and sensory analysis, 10 experts of this major were invited to taste the biscuits with different recipes. The sensory scores of the products were based on the four criteria of biscuits’ appearance, colour, flavor, and texture. Sensory evaluation results were described as x (mean) ± SD (n = 3) and overall quality on a 9-point hedonic scale [27]. Prior to taking part in the sensory evaluation of biscuits, participants were informed about the objectives of the research in order to make a well-informed decision as to whether or not they would like to participate. The approval was granted prior to the start of the study. Ethical approval was obtained from the Ethics Committee of the Second Affiliated Hospital of Henan University of Science and Technology (reference number 416527519). Written informed consent was obtained from the participants in this study.

### Composition analysis

Total sugar, moisture, fat, protein, and peroxide value were measured by the official AOAC analysis methods [28]. The total polyphenol were estimated by the Foline-Ciocalteau method [29]. Total dietary fiber content in the biscuits supplemented with nanocellulose was estimated according to the method described in the literature [30]. All analyses were carried out in triplicate. The results were expressed as the mean value, and the standard deviation was calculated.

### Animal experiment design

Kunming male mice (7–8 weeks old, average bodyweight 20–26 g) were purchased from Xi’an Fourth Military Medical University (Xi’an, China). Mice were randomly divided into three experimental groups and one blank control group according to their body weight. Each group comprised eight mice. The experimental groups are denoted as No.1 (low-dose group; mice were fed the biscuits with 5% nanocellulose), No.2 (medium-dose group; mice were fed the biscuits with 7% nanocellulose), and No.3 (high-dose group; mice were fed the biscuits with 9% nanocellulose). The mice in each group were fed pellets for three days to allow them to adjust to the environment. The temperature and humidity of the feeding environment were 18–25°C and 45–50%, respectively. Thereafter, the mice were allowed free access to food. The mice in the experimental group were given by gavage with a certain amount of nanocellulose-containing biscuits, which was provided in the form of a prepared solution. Each group was administrated by gavage once daily at 4 p.m. The doses for the three experimental groups were 10 times the recommended human dose (0.083–0.225 g/(kg·d)). The gavage volume was 1 mL/100 g body weight. Nanocellulose-containing biscuits were dissolved by warm water, and perfused. Mice in the blank control group were administered an equivalent volume of double-distilled water. The mental state, hair health, diet, water consumption, and urine of the mice were observed every day for seven weeks. The weight and food intake of each mouse were recorded every week before and after intervention. All animals feeding and experimental procedures were guided by the guidelines of Henan Provincial Experimental Animal Management Committee and approved by the Animal Ethics Committee of Luoyang Normal University and the Xi’an Fourth Military Medical University IRB (SCXK-(Military) 2012–0007).

### Data availability statement

The original contributions presented in the study are included in the article, further inquiries can be directed to the corresponding authors.

### Ethics statement

The animal study was reviewed and approved by the Animal Ethics Committee of Luoyang Normal University and the Xi’an Fourth Military Medical University IRB (SCXK-(Military) 2012–0007). Written informed consent was obtained from the owners for the participation of their animals in this study.

### Data processing

Data processing was implemented by using Excel 2013 and SPSS 22.0 (SPSS Inc, Chicago, IL, USA) and all analyses were performed in triplicate. Differences among groups were evaluated by one-way ANOVA, and *p*<0.05 was considered to be significant difference.

## Results and discussion

### Extraction of cellulose in waste chilli stems

**Fig 1:**
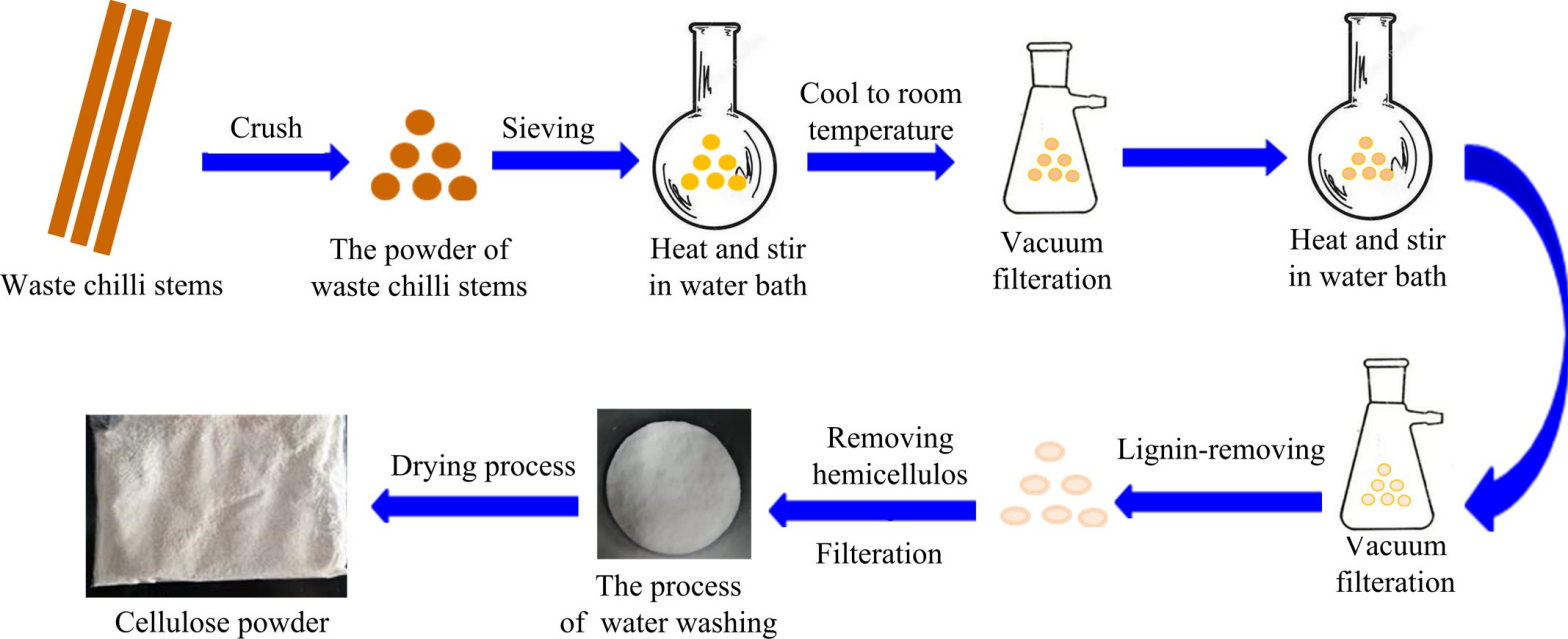
Extraction process of cellulose from waste chilli stems.

Extraction process of cellulose from waste pepper stems was shown in Fig 1. Ten groups of experiments were conducted, and the cellulose content of CS was determined to range between 15% and 34.5%.

The average cellulose content of the CS used in this study (27.4%) was lower than the average cellulose content of CS (37.27 ± 1.2%). This was attributed to chilli varieties, growth climate, sampling time, and experimental process [6–8]. Although the cellulose extraction rate in this study was lower than the current commercial cellulose extraction rate (≥99.90%), the cellulose extracted in this study could still be used as the raw material to fabricate nanocellulose.

### Preparation of nanocellulose

**Fig 2:**
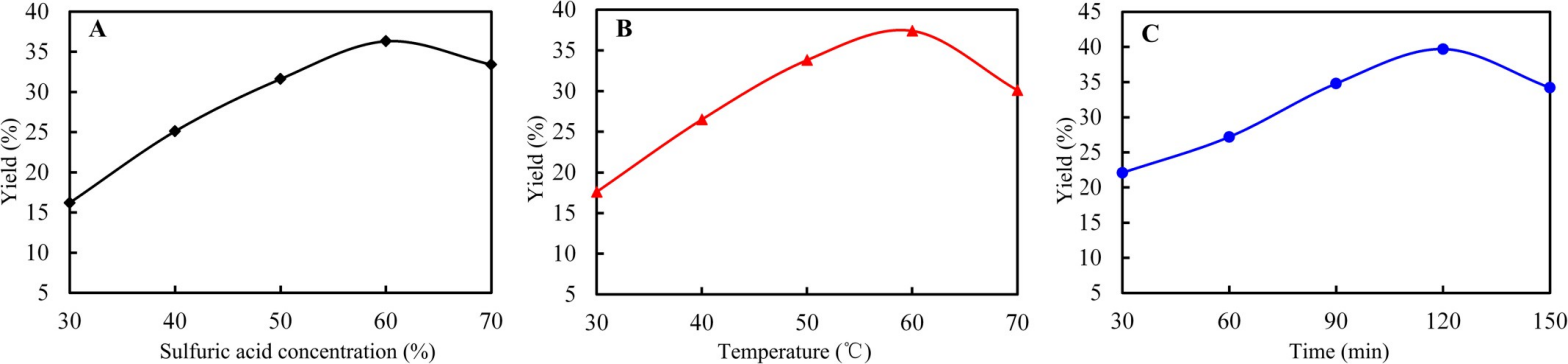
Optimization on preparation conditions of nanocellulose: (A) Sulfuric acid concentration, (B) reaction temperature, and (C) reaction time.

Cellulose comprises amorphous and crystalline regions. Nanocellulose was prepared using sulfuric acid, which removed the amorphous region and decreased the size of the crystalline region. In this study, the yield of nanocellulose was adjusted by controlling the concentration of sulfuric acid, reaction time, and reaction temperature. Cellulose samples were reacted with sulfuric acid under the same experimental conditions. Each experiment was performed in triplicate, and the average values were reported as the final values. As shown in (Fig 2).

For the experiments performed using sulfuric acid concentrations in the range of 30–60%, the yield of nanocellulose was directly proportional to the concentration of sulfuric acid (Fig 2A). The yield of nanocellulose was the highest for the experiment performed using a concentration of sulfuric acid of 60%. However, the study found that the yield of nanocellulose decreased with the increase of sulfuric acid concentration when the concentration of sulfuric acid in the system was greater than 60%. This was attributed to the acid hydrolysis of cellulose in sulfuric acid solution accompanied by swelling reaction [16, 31]. When the concentration of sulfuric acid was low (30%), the hydrolysis reaction was not sufficient due to the low swelling rate of cellulose, resulting in large particle size after hydrolysis [7, 13]. Therefore, the yield of nanocellulose was less. When the sulfuric acid reached 60%, the swelling rate of cellulose was good and the hydrolysis reaction was sufficient, so the yield of nanocellulose reached the maximum. When the concentration of sulfuric acid continued to increase, cellulose was over hydrolyzed in sulfuric acid solution to produce glucose, so the yield of nanocellulose decreased. When the concentration of sulfuric acid reached 70%, cellulose carbonization was serious. Therefore, the optimal concentration of sulfuric acid was 60%.

For the reactions performed in temperature range of 30–60*°C*, the yield of nanocellulose was directly proportional to the reaction temperature, and the yield of nanocellulose reached a maximum value for the reaction performed at 60*°C* (Fig 2B). However, the yield would decrease with the increase of temperature when the reaction temperature of the system exceeded 60*°C*. This was attributed to insufficient acid hydrolysis reaction at low temperature (30*°C*), such that the yield of nanocellulose was low. With the gradual increase of temperature, when the temperature reached 60*°C*, acid hydrolysis reaction was sufficient, which could promote the breaking of the glycosidic bond of cellulose molecules, reduce their degree of polymerization, and release more cellulose single crystals. However, when the temperature was too high (70*°C*), cellulose molecules were excessively hydrolyzed to produce glucose [13]. There might even be carbonization of cellulose molecules, so the yield of nanocellulose decreased. Therefore, the optimal reaction temperature was determined to be 60*°C*.

For the reactions performed for 30 and 120 min, the yield of nanocellulose was proportional to the reaction time (Fig 2C). Upon increasing the reaction time to 120 min, the yield of nanocellulose reached a maximum value. When the reaction time in the system exceeded 120 min, the yield of nanocellulose decreased with the continuous increase of time. This was attributed to sulfuric acid destroying an increasing number of hydrogen bonds of cellulose with increasing reaction time. With the increase of reaction time, the more hydrogen bond was destroyed, and the yield of nancellulose prepared by sulfuric acid was also increasing. When the reaction time was 150 min, sulfuric acid began to hydrolyze nanocellulose into polysaccharides, disaccharides, or monosaccharides, resulting in a decrease in the yield of nanocellulose [13, 27]. Therefore, the optimal reaction time was determined to be 120 min. In summary, the optimal reaction conditions were as follows: concentration of sulfuric acid of 60%, reaction temperature of 60*°C*, and reaction time of 120 min. The average yield of nanocellulose under these experimental conditions was 36.42 ± 1.36%.

### Characterization and analysis of cellulose and nanocellulose

#### Scanning electron microscopy analysis

**Fig 3:**
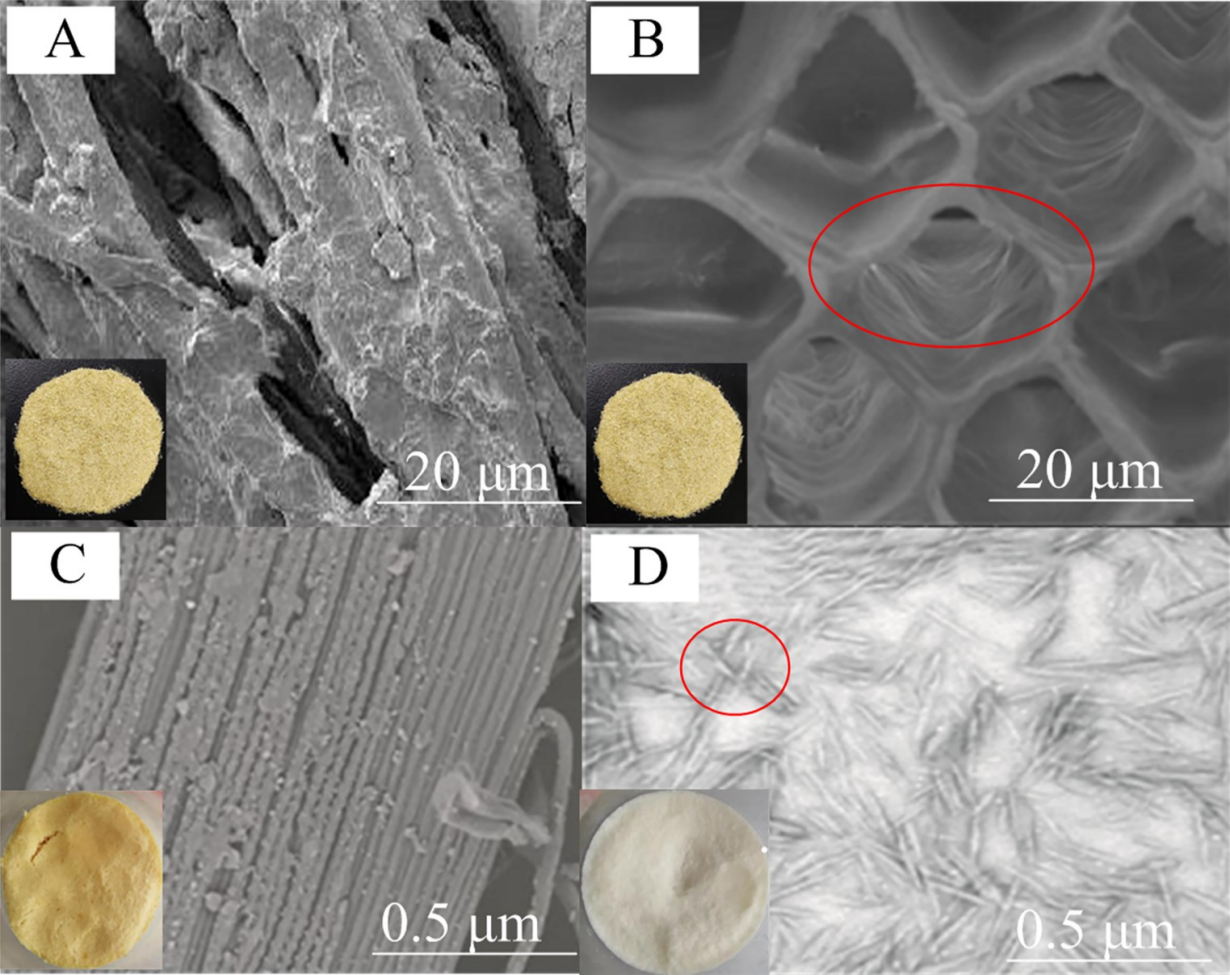
SEM images of waste chilli stems (A and B), cellulose (C) and nanocellulose (D).

The bundle cellulose structure of CS was not obvious because there was an external non cellulosic layer composed of cementation materials including lignin, hemicellulose, pectin, wax and oil (Fig 3A). It could be seen from Fig 3B that CS raw material itself had a spiral pore structure, as shown in the red circle of Fig 3B. After CS was treated with nitric acid–ethanol method, lignin, hemicellulose and other protective structures on the surface of CS were stripped, making the texture of the bundle cellulose structure clearer (Fig 3C). The nanocellulose prepared by sulfuric acid hydrolysis was mainly rod-shaped, small and uniform, with a diameter of about 10–30 nm and a length of about 150–300 nm. This was because sulfuric acid could effectively hydrolyze CS cellulose under appropriate conditions, reducing it from the original micrometer or even larger size to the nanometer size (Fig 3D). Nanocellulose had agglomeration phenomenon (Fig 3D, part in red circle). This was attributed to the fact that cellulose molecules contained a large number of hydroxyl groups. Because the particle size of nanocellulose was very small and the specific surface area was large, the contact area between nanocellulose particles increased [13]. Therefore, hydrogen bonds were easily formed in nanocellulose molecules, leading to the agglomeration of nanocellulose. In addition, there were a few other irregularly shaped nanocellulose with small particle size and no fiber bundle structure, which might be the result of further hydrolysis of nanocellulose by sulfuric acid [15].

### Infrared spectrum analysis

**Fig 4:**
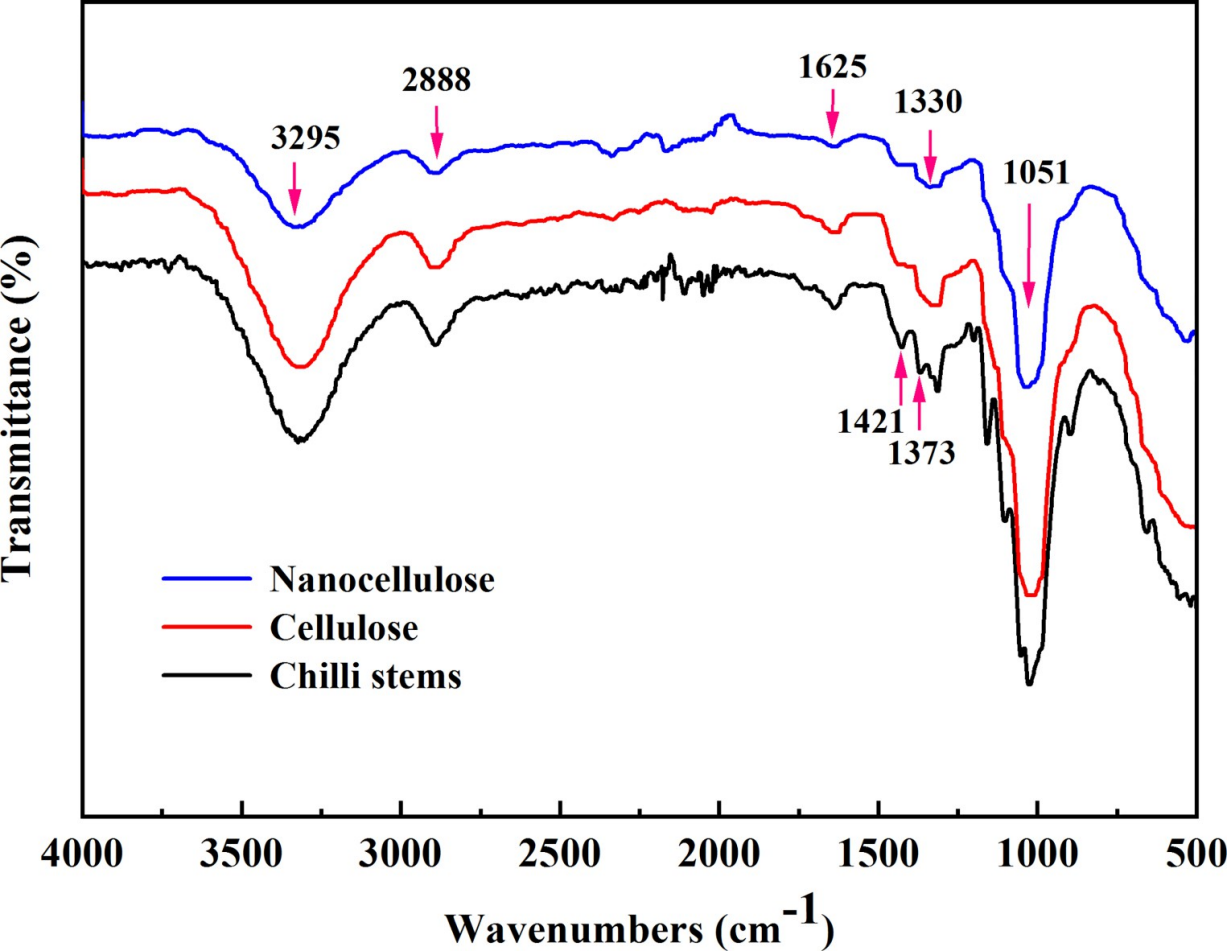
FTIR spectra analysis of cellulose and nanocellulose.

The infrared spectra of CS, cellulose and nanocellulose showed absorption peaks at 3295 cm^-1^, 2888 cm^-1^, 1625 cm^-1^, 1330 cm^-1^, 1051 cm^-1^. The absorption peaks at 3295 cm^-1^ were mainly due to the stretching vibration peak caused by hydroxyl–OH in cellulose and hemicellulose [32, 33]. As shown in (Fig 4). The peaks at 2888 cm^-1^ were attributed to the antisymmetric stretching vibration of saturated hydrocarbon.

–CH_2_ and stretching vibration of–CH in cellulose and hemicellulose. The absorption peaks at 1625 cm^-1^ was ascribed to the alkene C = C stretching vibration [15, 34]. The absorption peaks at 1330 cm^-1^ were probably–CH stretching of saturated hydrocarbons. The absorption peaks at 1051 cm^-1^ were caused by the stretching vibration of C-O-C-pyranose ring in cellulose [4, 5]. Compared with the cellulose spectrum, the characteristic peaks of nanocellulose spectrum had not changed significantly, and no new functional groups had been generated, which indicated that the nanocellulose prepared from cellulose in CS had not been damaged or changed in its chemical structure during the preparation process, and still maintained the basic chemical structure of cellulose molecules.

The difference in CS, cellulose and nanocellulose was that the absorption peak of CS at 1373 cm^-1^ was caused by the bending vibration of aromatic C-H and C-O rings of polysaccharides in cellulose. Compared with CS, the peak value of cellulose at 1421 cm^-1^ decreased, indicating that the total fiber content (the sum of cellulose and hemicellulose content) decreased. The increase of the peak value at 1051 cm^-1^ indicated the increase of its cellulose content, which indicated that its hemicellulose content decreased. The infrared spectra of nanocellulose and cellulose were similar. In addition, the decrease of the peak value of nanocellulose at 1625 cm^-1^ indicated the decrease of lignin content. To sum up, the content of cellulose and nanocellulose increased compared with CS. The content of hemicellulose and lignin decreased.

#### X-ray diffraction analysis

**Fig 5:**
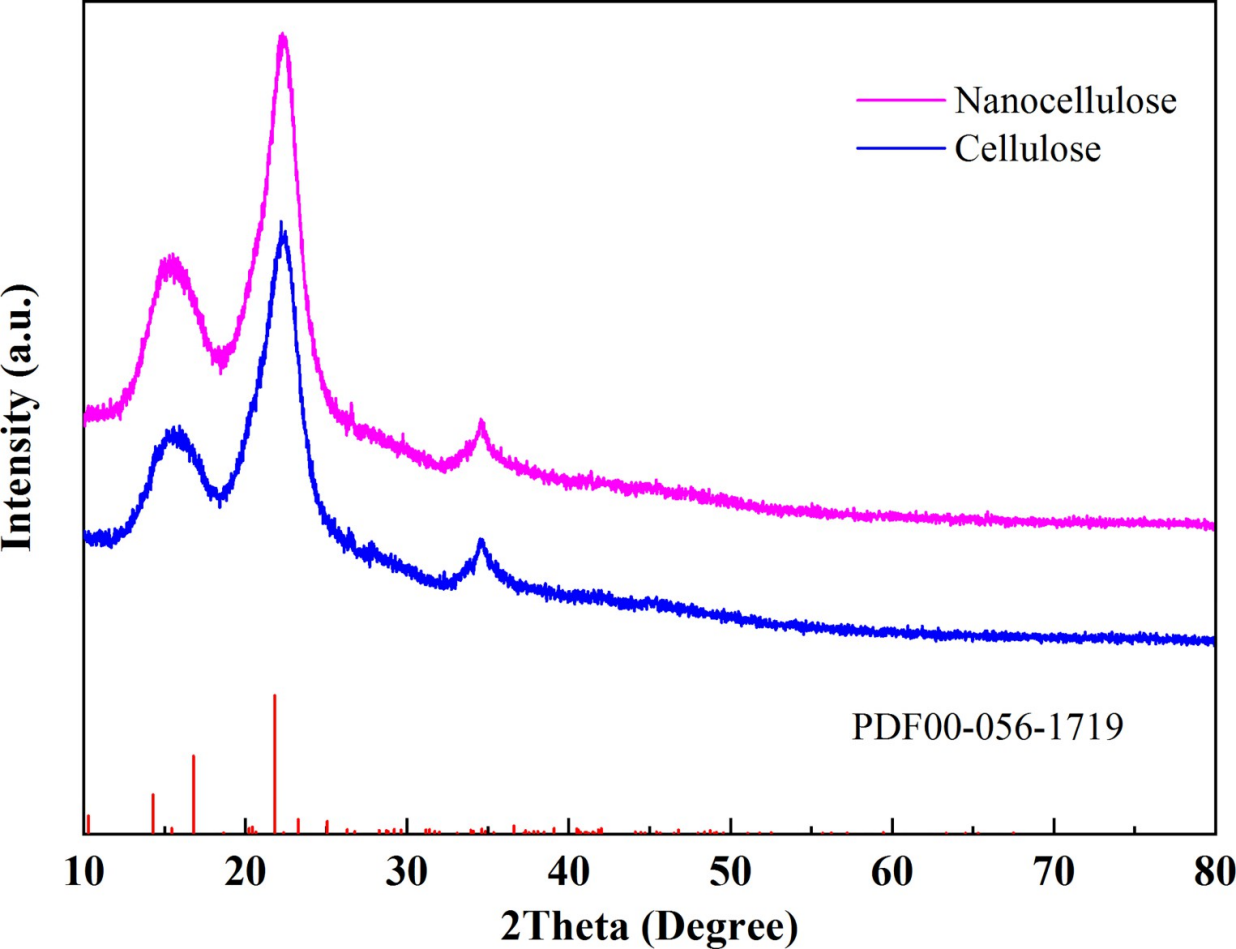
X-ray diffraction analysis of cellulose and nanocellulose.

The XRD spectra of cellulose and nanocellulose showed in Fig 5. The cellulose and nanocellulose showed weak diffraction peaks at 15.56°, 15.50°, 34.49 and 34.55°, and it belonged to the diffraction intensity of the amorphous region. The strongest diffraction peaks appeared at 22.18° and 22.29°, which belonged to the diffraction intensity of the crystal region. After treatment cellulose and nanocellulose were still type I crystal structures, which indicated that their crystal structures were not basically damaged [35]. The crystallinity was obtained by Eq (1). The crystallinity of cellulose extracted from CS was 50.78%, and the crystallinity of nanocellulose was 62.74%. The crystallinity of nanocellulose was higher than that of cellulose extracted from CS. This was attributed to H^+^ in sulfuric acid preferentially hydrolyzing the amorphous region of cellulose, which would degrade cellulose into nanocellulose and improve the crystallinity of nanocellulose. The crystallinity of cellulose and nanocellulose extracted with different materials and methods was different. Compared with the crystallinity of cellulose and nanocellulose extracted from rice straw (cellulose crystallinity 30–55%, nanocellulose crystallinity 35–45%) [15], wheat straw (cellulose crystallinity 35–65%, nanocellulose crystallinity 42.6–72.5%) [36], cotton (cellulose crystallinity 30–60%, nanocellulose crystallinity 48–64%) [20, 26] using liquid acid hydrolysis method, CS could be used as raw material to prepare nanocellulose with high yield and good performance. The research had certain feasibility.

### Sensory evaluation

A sensory evaluation of nanocellulose-containing biscuits was summarized in Table 2. The values are means ± SD of three independent determinations. With respect to colour, the score differed significantly between nanocellulose (7%) group and the control group (p<0.05). The values were 7.73 and 8.55, respectively. Compared with the control group, biscuits also became relatively hard with the increase of the level of nanocellulose. The hardness of biscuits might be due to greater water absorption capacity of the blends resulting in harder biscuits [23]. The appearance scores and colour scores showed a similar trend, which could probably be explained by the influence of the latter on the former to a certain degree. This was consistent with the result of a previous study [23, 37]. The flavour of biscuits was improved with adding of nanocellulose. The score was highest (8.27) when the content of nanocellulose was 7%. Then, the content of nanocellulose increased to decrease scores. Accordingly, the nanocellulose (7%) group was favourable to consumers. With regard to texture, increasing the content of nanocellulose leaded to a decrease in the fraction, which could probably be due to a decrease in size and an increase in surface roughness. When the nanocellulose was lower than 7%, the acceptability had no significant difference with the control group. The biscuits could be accepted by consumers. Overall, considering the colour, appearance, flavour and texture attributes, it could be inferred that the content of nanocellulose 7% was optimum. Thus, the biscuits of acceptable overall quality could be made using the content of nanocellulose 7% formulations, and the corresponding biscuits had a regular appearance and relatively smooth surface.

**Table 2:** Sensory evaluation of biscuits supplemented with nanocellulose.

Content of nanocellulose (%)	Colour	Appearance	Flavour		Texture	Acceptability
0	8.55±0.34^a1*^	8.46±0.16^a^	8.16±0.36^a^	7.72±0.63^a^		8.32±0.41^a^
5%	8.15±0.18^b^	8.27±0.58^b^	8.19±0.51^a^	7.68±0.32^a^		8.27±0.38^b^
7%	7.73±0.21^ab*^	7.76±0.24^a^	8.27±0.45^b*^	7.70±0.47^a^		8.31±0.22^a*^
9%	6.91±0.37^ab^	7.49±0.32^a^	7.63±0.62^a^	7.41±0.53^a^		7.73±0.62^ab^

^1^Values with different letters within the same column differ significantly (p<0.05).

* p <0.05 indicates a significant difference between groups.

#### Composition analysis of biscuits

The composition of biscuits with different nanocellulose content was shown in Table 3. The values are means ± SD of three independent determinations. The means followed by different superscript letters within a row are significantly (*p* < 0.05) different. The moisture, acid value and peroxide value did not differ significantly between nanocellulose groups and control group. The content of total sugar in biscuits supplemented with nanocellulose met the national standard (≤5 g/100 g), which belonged to low sugar food. Compared with control group, nanocellulose groups showed significantly lower (p<0.05) fat and total protein contents. When nanocellulose 7% was used, the levels of two components decreased 22.73% and 11.04%, respectively. The total dietary fiber content was positively correlated with different nanocellulose content. This was attributed to rich cellulose in CS. The foodstuff containing high fiber content might be given only if the product had at least 6 g of fiber per 100 g according to the claim that pointed out by regulation of the European Parliament of the Council (EC) [37]. Therefore, the biscuits supplemented with nanocellulose (>5%) could be considered as a high fiber content food.

**Table 3:** The composition of biscuits with different nanocellulose content.

Component (%)	nanocellulose addition level (%)			
	0	5	7	9
**Moisture**	2.23±0.35^a1^	2.31±0.43^a^	2.68±0.27^ab^	2.15±0.22^ab^
**Fat**	10.12±0.18^c^	9.56±0.26^b^	7.82±0.15^a^	6.76±0.48^b*^
**Total protein**	7.34±0.15^d^	7.12±0.21^d^	6.53±0.36^c*^	6.12±0.31^c^
**Total sugar**	2.23±0.01^d^	2.71±0.25^f^	3.53±0.32^b^	3.35±0.19^b^
**Acid value**	0.176±0.56^ab^	0.187±0.25^b^	0.192±0.63^a^	0.198±0.17^ab^
**Peroxide value**	0.049±1.21^f^	0.067±0.12^c^	0.062±2.31^d^	0.059±1.43^b^
**Total dietary fiber**	1.19±0.18^c^	6.58±0.32^e^	8.86±0.25^d*^	10.68±0.42^b*^

^1^Values with different letters within the same row differ significantly (p<0.05).

* p <0.05 indicates a significant difference between groups.

### Effect of nanocellulose on body weight and food intake of mice

**Fig 6:**
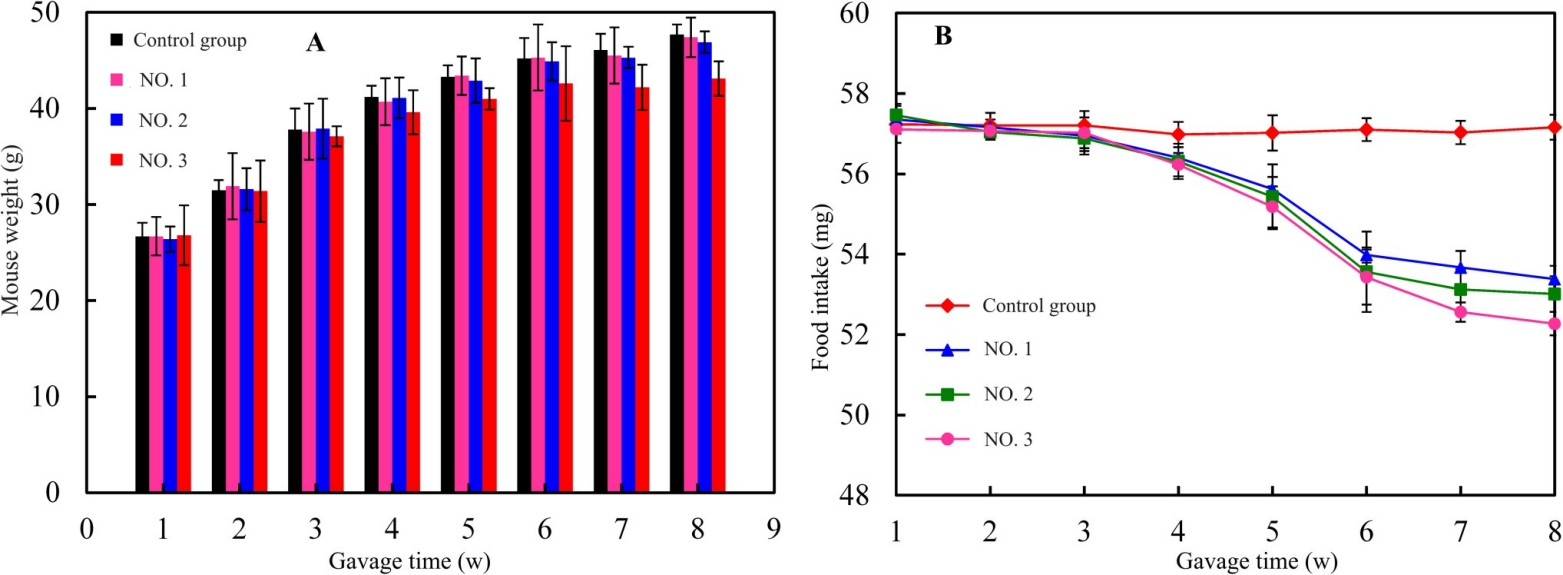
Effects of nanocellulose on weight gain and food intake in mice. (A) weight gain (g), (B) food intake (mg).

The mice in each experimental group ate normally, grew well, and did not experience diarrhea or other abnormal symptoms. Moreover, no deaths or infections occurred. The body weight of the mice in the four experimental groups increased gradually during the study (Fig 6A). Generally, after fat was ingested into the human body, the digestive enzymes in the intestine would decompose the triglycerides into fatty acids, and then the fatty acids would be absorbed by the intestine and reconverted into fat by the human body, which made people fat and gained weight. After seven weeks of sample administration, the body weight of the mice in the four experimental groups increased. The body weight of the mice in the high-dose group was significantly lower than those of the mice in the other groups. This was ascribed to the unique viscous properties of nanocellulose added to biscuits, which caused the mice to feel full. In addition, after mice ate the biscuits with nanocellulose, triglycerides were trapped in nanocellulose fibers, which reduced the efficiency of digestive enzymes involved in the decomposition of triglycerides. Therefore, it reduced the amount of fat that could be absorbed by mice, resulting in slow weight growth. The body weight of the mice in the high-dose group increased the slowest over the first week, this showed that the nanocellulose added to biscuits could significantly control the body weight of mice in the growth period. Starting with the third week, the average daily food intake of the mice in the high-dose group was lower than that of the mice in the control group (Fig 6B). These findings were consistent with the trends in the body weights of the mice illustrated in Fig 6A. Diets comprising high-protein and high-dietary-fiber foods can benefit people who like to lose weight and control their body weight. This may be attributed to high-quality proteins, such as whey protein, changing the metabolic mode of lipid molecules and promoting lipolysis [38]. In addition, dietary fiber forms highly viscous substances in the gastrointestinal tract to improve the viscosity of contents. The formed glue base can lower the gastric emptying rate and delay and inhibit the absorption of cholesterol, bile acids, glucose, and other substances [39]. Therefore, dietary fiber can effectively inhibit weight gain in mice. The results demonstrated that the intake of a certain amount of nanocellulose could significantly decrease the food intake of mice and inhibit their weight gain. Therefore, through this study, it could be seen that nanocellulose as a food additive or supplement was expected to be used to reduce the absorption of fat intake, reduce weight and control obesity.

## Conclusions

Nanocellulose is a new nano-material widely used in the world, with a wide range of applications from biological to non-biological fields. In this study, the cellulose content of CS ranged between 15% and 34.5% by using nitric acid–ethanol method. Nanocellulose was prepared using sulfuric acid hydrolysis method. The average yield of nanocellulose was 36.42% ± 1.36%. The structures of CS material, cellulose and nanocellulose were analyzed by SEM. The nanocellulose was mainly rod-shaped, with a diameter of about 10–30 nm and a length of about 150–300 nm. The nanocellulose prepared from cellulose in CS had not been damaged or changed in its chemical structure during the preparation process, and still maintained the basic chemical structure of cellulose molecules by FTIR spectrometer analysis. The crystallinity of nanocellulose was higher than that of cellulose extracted from CS, which were 62.74% and 50.78%, respectively. Acceptable overall quality of biscuits could be prepared using the content of incorporation of nanocellulose (7%) formulations, and the corresponding biscuits had a regular appearance, relatively smooth surface. The total dietary fiber content of biscuits was positively correlated with their nanocellulose content. Experiments performed on mice demonstrated that an intake of nanocellulose-containing biscuit significantly decreased the food intake of mice and inhibited their weight gain. The content of nanocellulose in biscuits was higher than 5%, which met the needs of modern “high dietary fiber” healthy food. Thus, it was found that nanocellulose could be used as dietary fiber in biscuits to improve their texture and taste and decrease the incidence rate of obesity without significantly affecting the quality of biscuits. Nanocellulose was completely extracted from CS of natural plants. Nanocellulose in the biscuits could improve the taste and nutritional value of biscuits. Once the product is launched, it will be very popular. However, pure natural cellulose is very safe, but the toxicological properties of nanocellulose need to be further studied to ensure its safety, which is also our next research content. These results provide a basis for subsequent studies on the efficient recycling of CS and application of nanocellulose as a food additive in the food processing.
